# Universities as catalysts of social innovation in health systems in low-and middle-income countries: a multi-country case study

**DOI:** 10.1186/s40249-020-00684-5

**Published:** 2020-07-11

**Authors:** Lindi van Niekerk, Don Pascal Mathanga, Noel Juban, Diana Maria Castro-Arroyave, Dina Balabanova

**Affiliations:** 1 The Bertha Centre for Social Innovation and Entrepreneurship, University of Cape Town Graduate School of Business, Cape Town, South Africa; 2 London School of Hygiene and Tropical Medicine, London, UK; 3 The Special Programme for Research and Training in Tropical Medicine (TDR), Geneva, Switzerland; 4 University of Malawi, College of Medicine, Blantyre, Malawi; 5 University of the Philippines, School of Medicine, Manila, the Philippines; 6 Centro Internacional de Entrenamiento e Investigaciones Medicas (CIDEIM), Cali, Colombia; 7 ICESI University, Cali, Colombia

**Keywords:** Social innovation, University, Healthcare delivery, Health system, Low-and middle-income countries

## Abstract

**Background:**

Social innovation (SI) in health holds potential to contribute to health systems strengthening and universal health coverage (UHC). The role of universities in SI has been well described in the context of high-income countries. An evidence gap exits on SI in healthcare delivery in the context of low- and middle-income countries (LMICs) as well as on the engagement of universities from these contexts. There is thus a need to build capacity for research and engagement in SI in healthcare delivery within these universities. The aim of this study was to examine the adoption and implementation of network of university hubs focused on SI in healthcare delivery within five countries across Africa, Asia and Latin America. The objectives were to describe the model, components and implementation process of the hubs; identify the enablers and barriers experienced and draw implications that could be relevant to other LMIC universities interested in SI.

**Methods:**

A case study design was adopted to study the implementation process of a network of university hubs. Data from documentation, team discussions and post-implementation surveys were collected from 2013 to 2018 and analysed with aid of a modified policy analysis framework.

**Results/discussion:**

SI university-based hubs serve as cross-disciplinary and cross-sectoral platforms, established to catalyse SI within the local health system through four core activities: research, community-building, storytelling and institutional embedding, and adhering to values of inclusion, assets, co-creation and hope. Hubs were implemented as informal structures, managed by a small core team, in existing department. Enablers of hub implementation and functioning were the availability of strong in-country social networks, legitimacy attained from being part of a global network on SI in health and receiving a capacity building package in the initial stages. Barriers encountered were internal institutional resistance, administrative challenges associated with university bureaucracy and annual funding cycles.

**Conclusions:**

This case study shows the opportunity that reside within LMIC universities to act as eco-system enablers of SI in healthcare delivery in order to fill the evidence gap on SI and enhance cross-sectoral participation in support of achieving UHC.

## Background

Complex systemic global challenges have seen a growing interest in social innovation (SI). SI has emerged as a way of understanding and producing lasting social change when current systems and structures fail [1]. As a socially and politically constructed concept, scholars have described two paradigms of SI: the technocratic and the democratic [2, 3]. The instrumental technocratic paradigm understands SI within the context of the neoliberal political agenda, focusing on solutions that can create greater efficiency gains in order to solve the crises of the welfare-state and thus is concerned with models such as social enterprises [4, 5]. This frame is predominant in high-income countries (HIC) such as the United Kingdom and Europe [6–8]. In contrast, the democratic paradigm adopts more emancipatory aims: meeting human needs, raising the participation of the marginalized groups and empowering citizens through greater access to resources and social and political capacities. Montgomery et al. (2016) warns that the technocratic paradigm could result in the creative destruction of social relations, by reinforcing vertical distributions of power within society, while the democratic paradigm gives rise to the possibility of creative transformation of social relations [3, 9]. The democratic paradigm holds most promise for low and middle-income countries (LMICs), especially as overcoming power hierarchies and fostering inclusion of previously excluded groups can be a catalyst towards whole system transformation. In this paper concerning LMICs, the democratic perspective is taken and SI is understood as per Westley’s (2010) definition: “*Social innovation is a complex process of introducing new products, processes or programs that profoundly change the basic routines, resource and authority flows, or beliefs of the social system in which the innovation occurs*” [10].

The achievement of Universal Health Coverage (UCH), a target of Sustainable Development Goal 3, remain a major challenge as half the world’s population still lack access to basic essential health services [11]. Despite impressive scientific and technological innovations to improve disease control and treatment, disparities in wealth and health have widened [12]. The challenge of equitable healthcare delivery is an opportunity for SI [13]. Contrary to the traditional expert driven top-down approaches used in health, SI emphasises the bottom-up design and implementation of interventions and enables the participation of grassroots actors. Collectively, these actors contribute to knowledge construction, redistribution of power horizontally and empowerment, and agency through increased socio-political capacities [3] Now more than ever, LMIC health systems are in need of delivery solutions that are developed through participatory action, embedded in the local communities and appropriate to the country context [14].

Universities have emerged as institutional structures that play a key role in enabling and supporting SI in different ways [15]. Traditionally, universities have been regarded as elitist institutions accessible to only a small portion of the population, focused on delivering three types of services: research, education and societal interaction with industry, government and communities [16]. Universities have a long legacy of pioneering in scientific and technical domains. However, in a parallel trend, universities have started recognising their unique role in contributing to societal wellbeing through addressing prominent social challenges. The extensive resources, research expertise and connections of universities hold significant potential to support SI [15–18]. Benneworth et al. (2015) provides a typology of universities’ contributions to SI, either as knowledge providers or knowledge bridges; as providerss of material resources such as finances in support of the testing or scale up of innovations or physical space; and in acting as advisors or mentors to social innovators [15]. McKelvey (2018) conceptualises the role of the university as intermediaries in delivering SI through ‘academic engagement with society’ [16]. Practically, this has translated into universities developing dedicated courses on SI including student community-internships, engaging in research at various points of the SI lifecycle and the creation of dedicated ‘innovation laboratories’ where stakeholders can unite in solution creation or community-campus collaborations. Yet, authors recognise that the most valuable contribution universities can achieve is to incorporated SI as part of their broader university-wide strategic goals [15, 17, 19]. To this extent, Matheson (2008) states that universities need to revise internal processes and reward systems to promote such cultural shifts within the academic domain [17]. Literature focusing on LMIC university engagement in SI is sparse.

In this paper, we present a case study on the adoption and implementation of a network of university hubs focused on SI in healthcare delivery in five LMICs across Africa, Asia and Latin America. The hubs were established in response to the evidence gap on SI in healthcare delivery in LMICs, as studied through a health systems and policy lens. The dearth of academic publications about the subject however does not imply an absence of SI but can be explained by the limited engagement of LMIC universities. Significant investments have been made by LMIC universities to strengthen their research capacity and their outputs have been closely linked to support the achievement of national health plans [20, 21]. These universities are also strategically equipped as a bridge between communities, government and other country actors. Thus, the opportunity to fill the evidence gap on SI by leveraging the existing research capacity and builing technical capacity of these universities to engage in SI in health.

## Methods

### Study design

The case study remains one of the most commonly employed study designs in SI due to its exploratory and explanatory potential [22, 23]. Case studies are methodologically an example of ‘researching ‘open systems’ where the phenomena can less be controlled, variables are not linear and they interact in changing ways over time’ [24]. Just like SI itself is an ongoing evolving process that is highly context bound, the establishment of the hubs were in and of themselves regarded as an innovative endeavour. It was a new structure to introduce a new concept in a predefined setting and its success was influenced by its adaptation to the local contextual realities. The phenomenon of study was the process and actions by which SI was adopted as part of university institutions in LMICs over a period of time. Recognising that ‘hubs’ in the context of the innovation are often known as dedicated physical spaces, we borrowed the term to best describe a fledgling informal structure to initiate activities around SI within the university and beyond.

### Country selection

The country selection for inclusion in this case study was done purposefully, based on countries involved in a project called the Social Innovation in Health Initiative (SIHI). SIHI was launched in November 2014 through the financial and technical support from the Special Programme of Research and Training in Tropical Diseases. A common factor across all implementing institutions was that at the time of establishment, SI was not a well-recognised or researched phenomenon in health delivery and hence they were awarded a grant to these institutions become a part of the SIHI network of university hubs.

### Data collection and analysis

For this case study, data was collected from several sources including historical operational documents, proposals and reports, minutes from ongoing partner implementation discussions and a post-implementation hub survey spanning the period from 2013 to 2018. Data was collected in an ongoing manner by the core project implementation team as this was an emergent and evolving process, not pre-defined at start but rather based upon needs, resources becoming available and presenting opportunities. Data was thus analysed retrospectively drawing on Walt and Gilson’s policy analysis framework that incorporate concepts of content, the process, the context, the actors [25], but also identifying additional key themes such as culture and mindset. From this analysis we are able to draw the implications that establishing a SI hub could hold for other universities located in LMICs.

## Results

**Fig. 1:**
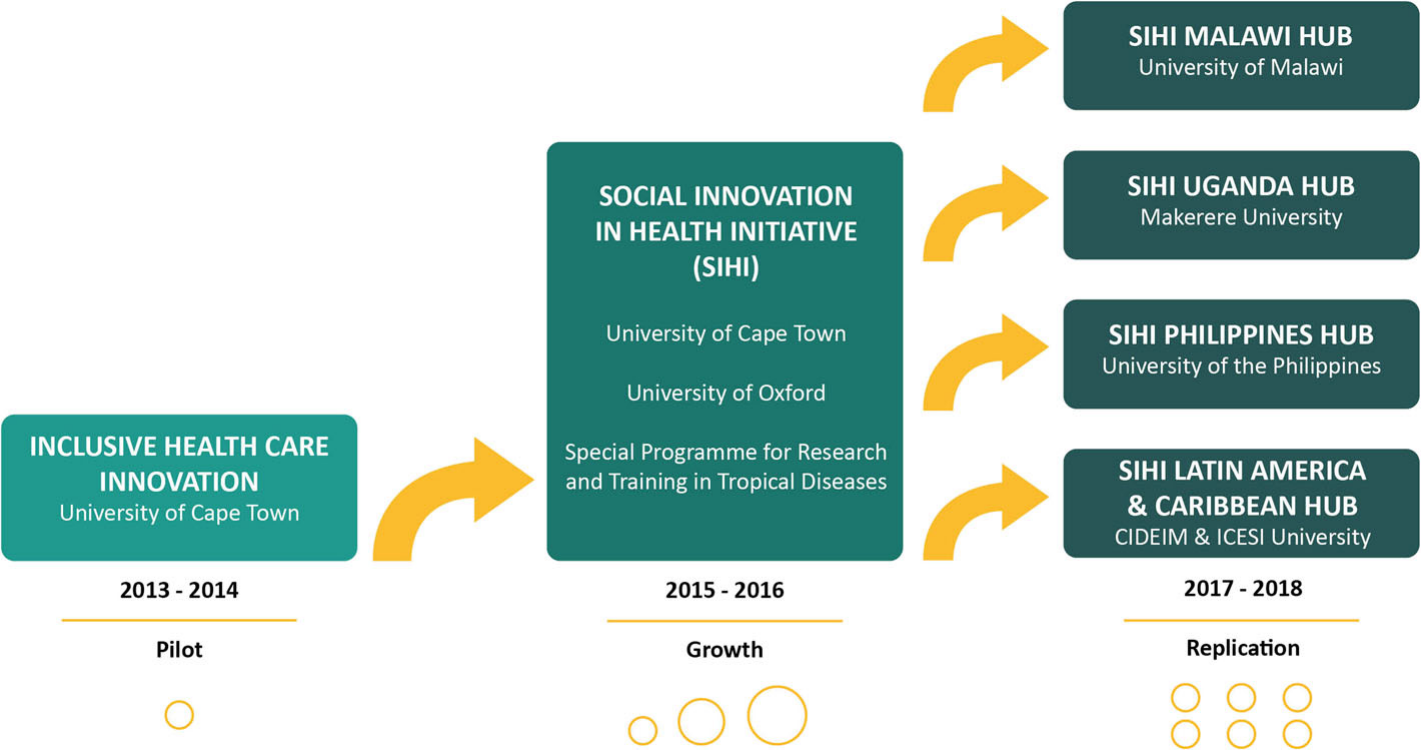
Development of university-based social innovation hubs

We first describe the process of development of university-based SI hubs and their functioning over a period of 5-years. This includes the sequence of the steps: from piloting the first hub in South Africa, its expansion and replication in four other countries (Fig. 1). In this section, we describe the features of the hub model, its constituent components and activities, the implementation process and actions as well as the outputs that were associated with their functioning. We conclude with an analysis on the enablers and barriers relevant to this process in a LMIC university context.

### Hub model: key features, mindset and culture

The intentions inherent in the development of the SI in health hub model were three-fold. Firstly, although wanting to learn from similar university hub models developed in HIC settings, it was a key intention not to merely replicate such a model but to develop a model appropriate and contextually relevant to LMIC settings. Secondly, in each of the institutional settings resource constraints were a reality and thus it was deemed more feasible to start with an informal ‘hub’ structure embedded within an existing department, and so doing leveraging the human and capital resources already present. Thirdly, in line with the SI process the intention was not to prescribe but to allow for evolution and adaptation by giving implementing country institutions the freedom to make alternations to the model over time.

The model evolved over the period of five years, first piloted in South Africa. The first pilot hub was launched in 2014 at the University of Cape Town (UCT) Graduate School of Business (GSB)’ Bertha Centre for Social Innovation and Entrepreneurship. The hub was launched under the name *Inclusive Healthcare Innovation*. It was established as a cross-faculty collaboration between the UCT Graduate School of Business and the UCT Faculty of Medicine. Prior to launching the hub, the team conducted a strategic scoping exercise of the South African health system to identify gaps and opportunities where SI could contribute. Four gaps in the local eco-system were identified: a) innovation in health is synonymous mainly with scientific, technology or medical discoveries only; b) cross-sectoral and interdisciplinary collaboration were limited, resulting in multiple silos across the health system with limited collaboration; c) minimal opportunity existed for participation of frontline actors and civil society in innovation development to support of health system strengthening; and d) a pervasive low morale and hopelessness existed among actors, especially those working in the public sector. They regarded health systems change too difficult to achieve in the context of increasing service demand and scarce resources and that they did not have the power to affect change themselves. These gaps were not unique to South Africa, but they were representative of other LMIC contexts.

The mindset and culture embodied within this pilot hub, later replicated in other hubs, was based on the SI heuristic as developed by Nilsson et al. (2015) (See Table 1) [26]. This heuristic embraces a transformative paradigm, in contrast to the corrective or problem-solving paradigm often adopted in health care, where problems are analysed as abstract phenomena devoid of human influence, incremental changes are designed to ‘fix what is broken’, and only then are others brought on board. As articulated by Peter Block: “*Problem solving can make things better but it cannot change the nature of things, especially systems which are complex and adaptive* [27].”

**Table 1:** Heuristic comparison [26]

Problem-Solving Heuristic (Corrective paradigm)	Social Innovation Heuristic (Transformative paradigm)
Problems	Possibilities
Gaps	Strengths
Command & control	Co-creation
Implementation	Evolution
Products & Processes	Patterns
Externalized institutions	Internalised institutions
Despair	Hope

Based on the gaps identified in the strategic scoping exercise and drawing on the SI heuristic, the implementing team adopted four values inherent to the pilot hub model. These values remained core to all the subsequent hubs.

- Inclusion – The hub was to act as a platform to unite four health system actor groups across sectors, disciplines and levels: academia (faculty and students), government (policy makers and front-line workers), social innovators (non-state actors) and other health implementers (for profit or not-for-profit health implementers). The hub positioned itself as the interface between the university and the health system and through its activities, it opened up opportunities for wider engagement and collaboration.
- Asset-based – Instead of focusing on deficits and shortcomings within the health system, hub members aimed to identify the strengths, contributions and existing assets within the health system. Starting by highlighting assets and not problems was key to gaining buy in and achieving a more possibility-focused orientation.
- Co-creation – Diverting from a top-down command-control approach, the hub focused on creating with actors, especially those at the frontline or grassroots level, as participants within the system or as recipients of care. It recognised their inherent value, perspectives and contributions they can bring to strengthening the system.
- Hope – SI calls for new visions to recognise what might be, even before it exists and despite what is currently practiced. This vision is inspired by hope that unlocks new creativity. Hope was a core quality to overcome the despair derived from failed attempts to shift bureaucratic procedures associated with the health system.

The practical application of these values in the activities of the hubs are illustrated in Table 2.

**Table 2:** Hub components and mechanism overview, rationale and leverage

Core component	Mechanism	Description	Actors engaged	Rationale	University leverage
**Research**	Crowdsourcing contests Case Study Research	A public contest soliciting applications for social innovations. Qualitative case studies on selected social innovations identified in the crowdsourcing call. Rich descriptions of each social innovation’s components and drawing lessons of health systems relevance.	Social innovators Academics Government – policy makers	Conducting a review of published literature, revealed very limited examples on social innovation in health. Thus, to identify these grassroots projects, it was necessary to solicit it directly individuals and organisations working at grassroots level. Case examples of social innovation formed the foundation for all further discussion, especially in a context / setting where social innovation is not yet a well understood phenomenon. Descriptive case studies assisted in generating first-level evidence to highlight the presence of social innovation and the potential contribution it could make. These cases assisted in identifying areas of further research.	Universities are associated with new knowledge creation and have research expertise in multiple fields to draw upon. Social innovation research requires multi-disciplinary engagement. Hubs are regarded as trustworthy platforms for innovators to share their work with, for the purpose of developing shared knowledge and learning that could benefit the health system as a whole.
**Community-building**	Convenings and communication	Conferences, meetings and workshops hosted to share research findings and showcase social innovation and invite collaboration on developing a shared future agenda. Regular newsletters and personal communication.	Social innovators Academics (faculty & students) Government (policy makers & frontline workers) Health implementers (not-for-profit & for-profit)	Fostering cross sectoral linkages with multi-sectoral actors engaged in social innovation at a national and international level, including the research participants. All gatherings were hosted taking into consideration the mindset and culture – thus creating spaces for sharing and discussion where all voices have equal value. All spaces chosen for gatherings non-traditional meeting venues and detailed attention was paid to create a different experience for participants, that transcends content.	Universities are traditionally not-for-profit and non-politically affiliated. Hubs were able to act as a neutral convener of different sectors, sharing the lessons and learning widely and facilitating dialogue across levels of the health system and sectors.
**Storytelling**	Film & media	Videos on social innovations. Thematic short film. Positive hope-filled photography.	Social innovators Academics (faculty & students) Government (policy makers& frontline workers) Health implementers (not-for-profit & for-profit)	Visual media using a storytelling approach transcended disciplinary mental confines to first and foremost create a shared human experience. Stories enabled the viewer to personally relate and identify a truth for themselves. It created greater engagement in the research content and an openness to participate.	Universities have access to different skills expertise. The pilot hub was able to train two staff members on film making and photography.
**Institutional embedding**	Cross-organisational activities Engaging leaders	Activities to engage participation of other departments, or champions within an institution.	Heads of Department Deans, Vice-chancellors	To institutionalise social innovation beyond the hub into other areas of the organisations / institutions engaged.	Universities are able to bridge the divide between the grassroots and the top-levels of health decision-makers. By fostering relational engagement between them, social innovation goes transcends beyond theory to practical application at various levels of organisations and institutions, irrespective of their specific disease focus.

### Hub components

During 2013–2014, the pilot hub in South Africa started two work components that attracted further support in order to be scaled up: i) research to identify and study social innovations in health and ii) a multi-actor community of individuals and organisations interested in SI in health. At the end of 2014, the pilot hub was awarded a research grant to extend this work beyond South Africa. The scope of the research was extended to identify and study social innovations across the Africa, Asia and Latin America region. Through this research grant, a new collaborative partnership—the *Social Innovation in Health Initiative (SIHI) -* was established between the University of Cape Town, the Oxford University Skoll Centre for Social Entrepreneurship and the Special Programme of Research and Training in Tropical Diseases (TDR, also the funder).

In addition to research and community building work, storytelling was added as a third component and this was an important addition to the growth of the hubs. Research of each SI included photography and film, enabling the research to be shared not only in an academic format but also in a format that was relatable and accessible to a non-academic audience. Storytelling served as key to transcend factual interpretations, as it opened the space for a relational engagement between actors. During identification and data collection, grassroot actors and social innovators regularly communicated to share stories from the field. A series of stakeholder meetings, based on design-thinking methodology [30] and organizational development methodology [27], were hosted at a global level to unite the four main actor groups under one roof and facilitate them through a process of sharing and collaboration. TDR, as funder and partner, played an influential role in attracting other international agencies to these meetings and in gaining their commitment their endorsement of SI in health.

The fourth work component, institutional embedding, was achieved by engaging grassroots actors and social innovators as collaborators rather than mere research participants. This supported the embedding of SI within international agencies such as the World Health Organization and funding bodies such as Swedish International Development Corporation Agency. Social innovators included actors working at grassroots level from government, non-governmental or civil society organisations, private companies or universities. Significant time investment was made to build strong relationships with these actors throughout the research, community-building and storytelling activities. For these organisations, hearing a first-hand account from the research participants (the social innovators) and witnessing the reality of the context in which they worked (by watching the films) unlocked further resources in support of country hub replication.

**Fig. 2:**
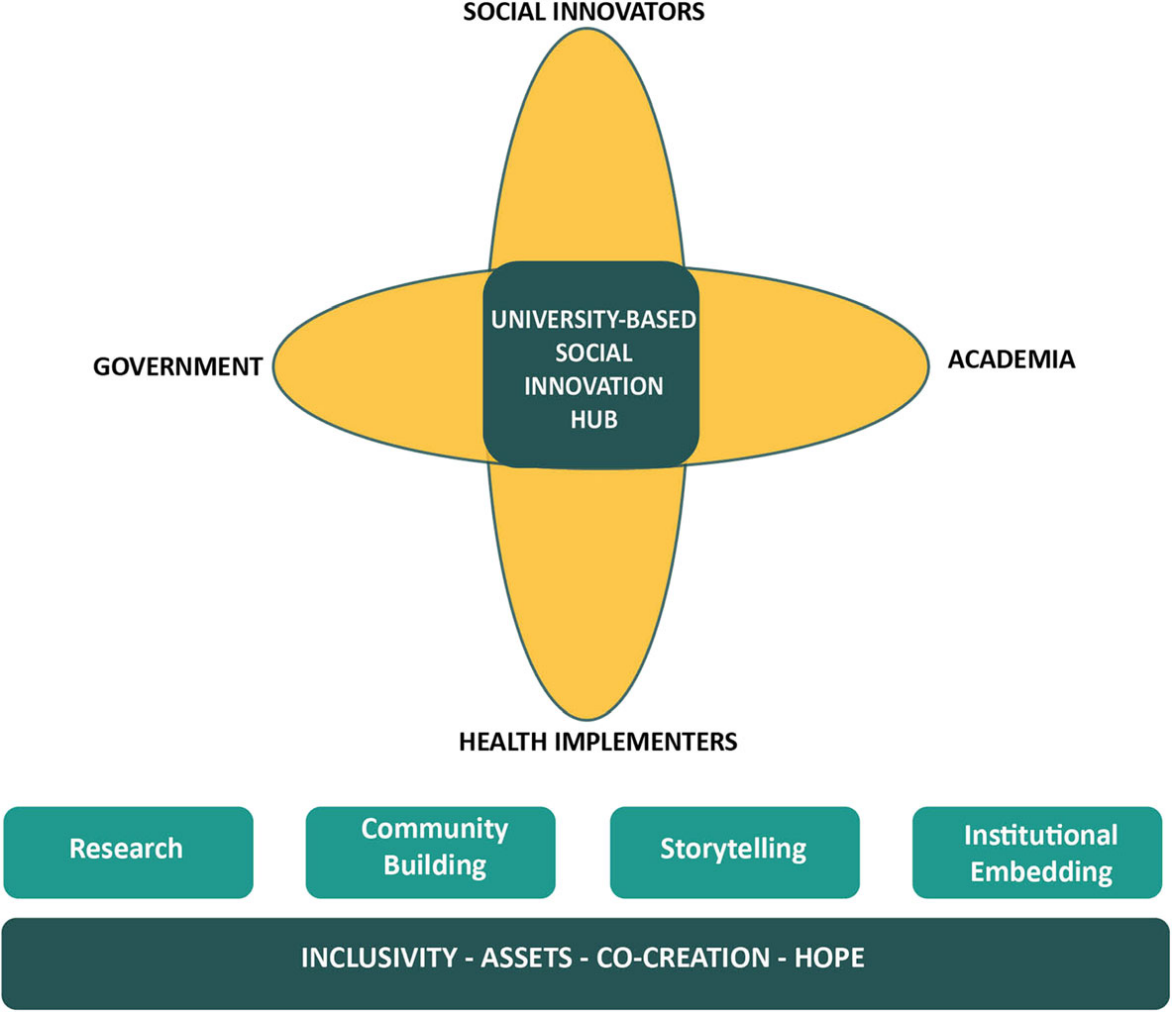
University-based social innovation hub model

The experience from 2013 to 2015 assisted in identifying the four core work components of a university-based hub for SI in health systems (See Fig. 2). Table 2 provides a more detailed explanation of the various mechanisms and rationale behind each of the four work components

### Implementation process: towards multi-country hubs

From 2017 to 2018, efforts were initiated to take the learning gained from the pilot and growth phase in order to apply it to the replication of the hub model in four other LMIC universities: the University of Malawi, Makerere University, University of the Philippines and at Centro Internacional de Entrenamiento e Investigaciones Medicas (CIDEIM) in collaboration with ICESI University. The intention was for each hub to operate independently while sharing their implementation experiences among each other. The hub replication was led by the South Africa team in partnership with TDR.

Contrary to university hubs in many high-income countries, the goal of these hubs was not to develop new social innovations but to act as catalysts to embed SI and research in SI in support of the local health system policy and practice. These universities and research institutions were uniquely positioned for this role for three reasons. Firstly, each university were highly regarded research institutions in their respective countries. They were trusted by government to provide rigorous and reliable evidence-based recommendations. Secondly, each hub team had access to a diverse range of disciplinary expertise from across its faculty. SI research required greater participation of social scientists. Thirdly, each university had well established social networks and relationships with government, non-governmental organizations, international agencies and even some private sector organisations. It was thus easy for them to become a convening platform for different SI stakeholders across sectors.

The approach adopted by the replication team, in supporting the establishment of these new hubs, were in line with the SI heuristic (Table 1). Concentrated effort was made not to impose expertise but to unlock the already existing capacity within the partner universities, and to complement this with the lessons learned in earlier years. Being hopeful, supportive and positive in all interactions with the partner universities was key, especially as they frequently encountered initial resistance to introducing a new concept within an established system. All partners implemented the same set of activities in the initial 2 years, as part of learning the knowledge and skills associated with SI. The hubs operationalised their core values throughout their various activities as explained in Table 3.

Hubs were not envisioned to become permanently sustained structures but rather a core focus was on institutionalisation of SI in health as an area of exploration within each university and country. Institutionalisation was to be achieved in four ways: a core team, a strategic and operational plan, partnerships and leveraging resources.

Initially hubs each hub was led by core team dedicated to the task (Table 4). Within each institution, funding support made it possible for identified hub leads to appoint at least two dedicated junior staff members to support the activity implementation. The attitude and mindset of those involved were important in the selection of staff as pioneering a concept that has not yet been recognised within the health domain nor at international or national level requires strong commitment and an appetite for risk and entrepreneurial spirit of creativity. The hubs all attracted young professionals as project managers and coordinators, who with senior guidance and empowerment, were able to show strong leadership and initiative to ensure their hubs are successful.

**Table 3:** Social innovation hub values and application

Value	Practical application in hubs (Examples from 2014 to 2018)
**Inclusion**	The launch of the pilot hub South Africa coincided with the first Inclusive Healthcare Innovation Summit (organised by the hub as its first activity). The status quo for health-related meetings in South Africa was attendance by health professionals and experts. This Summit was opened up to any individual or organisation with an interest in innovation in health delivery. It attracted 284 delegates from across 8-sectors. Delegates included among others: policy makers, researchers, frontline health workers, entrepreneurs, non-governmental organisation workers, and students. A delegate comment: “*you really didn’t rent the usual crowd”* [28]
**Asset-based**	All country hubs engaged in recognising and promoting existing social innovations in their settings in order to illustrate that despite health system challenges faced, there are also an abundance of unknown, yet impactful, solutions at grassroots level.
	To do so, crowdsourcing contests were done to identify positive examples of social innovations and these innovations were awarded and promoted. In the case of Latin America hub, their social innovators were recognised by the Pan American Health Organization (PAHO) and in the Malawi hub, the innovators were recognised by the Malawi Ministry of Health. Research was done on these cases to build the evidence base on the application of social innovation in health systems.
**Co-creation**	In 2018, all country hubs commenced a hub-strategy development process. This was conducted thoughtfully and purposefully, first through stakeholder mapping and then engaging all the relevant stakeholders in informing the strategy. This included stakeholders from the Ministry of Health but also grassroots social innovators. In the Philippines, the hub created a strong partnership with the national health research development council which lead to the co-creation of a national social innovation identification process and annual award.
**Hope**	Language and design played an important role in fostering a renewed sense of future possibility, despite resource constraints. A communication campaign was launched in lead up to the 2015 the global convening on social innovation in health. The campaign used the slogan “WHAT IF…” eg. *WHAT IF healthcare delivery in low-income countries could be re-imagined*?” All communication products were designed according to a specific brand that was created. Core to the brand is displaying images showing strength, courage and vibrancy of people living in low and middle-income countries. Relational engagement in the form of cross-cutting nature of in-person meetings and workshops, facilitation techniques to generate discussion between individuals from opposite spectrums further supported fostering hope [29].

This small core team became responsible for driving implementation while continuing to pursue partnerships with key national or regional organizations (Table 5). Each partner university was encouraged to let its hub evolve, based on available opportunities and resources, and to co-create its own contextually relevant hub strategy with its core stakeholders and partners (social innovators, academics and government representatives). The Malawi hub hosted several workshops with their key stakeholders to inform their strategic approach and tailor the design of their activities accordingly. The hub representing the Latin America and Caribbean (LAC), decided to develop a regional strategic focus, engaging neighbouring countries. This was supported by their strong regional partnership with the Pan American Health Organization (PAHO). The regional geographic focus of this hub did pose a challenge to build the momentum initially required to attract the participation of actors in its activities. Yet over time, the momentum was gained despite it requiring more time investment. Aligned to its regional strategy, the LAC hub also established a cross-country stakeholder community of eight participating universities. With the support of researchers and professors from these universities, the hub offered courses and workshops on SI in health and community-based-participatory methodologies. This community has enabled research collaborations to be fostered between participating universities and research on SI in health to be embedded in other universities. In the Philippines, the hub was successful in having SI in health taken up as the theme of the annual conference of the Philippines Council for Health Research. This supported the institutionalisation of SI at a national level and led to the creation of a national award, the Gelia Castillo Award for Social Innovation in Health, to be created in partnership with Philippines Council for Health Research and Development (Department of Science and Technology) and the Department of Health. Through partnerships established the hubs were able to leverage in-kind technical and also financial resources to support it.

**Table 4:** Hub positioning and staff composition

Hub	Housing department	Core staff composition
SIHI Malawi -University of Malawi, College of Medicine	Malaria Alert Centre	Hub Lead (MBBS, MPH, PhD)
		Project Manager (BA Community Health, MPH)
		Project Coordinator (BA Journalism)
SIHI Uganda -Makerere University, School of Public Health	Department of Community Health & Behavioural Sciences	Hub Lead (MBChB, MSc Int Health, PhD)
		Project Manager (MPH, BA Economics)
		Project Coordinator (BSc Enviromental Health)
SIHI Philippines –University of the Philippines Manilla, College of Medicine	Department of Epidemiology	Hub Lead (MD, MSc Epidemiology)
		Project Manager (RN)
		Communications Coordinator (MSc Communication)
SIHI Latin America and Caribbean –CIDEIM in partnership with ICESI University	Centro Internacional de Entrenamiento e Investigaciones Médicas (CIDEIM)	Hub Lead (PhD)
		Lead of Research (PhD)
		Project Manager (B Pharm)
		Post-doctoral Researcher (PhD)

*BA* Bachelor of the Arts; *B Pharm* Bacelor of Pharmacy; *CIDEIM* Centro Internacional de Entrenamiento e Investigaciones Médicas; *MBBS / MBChB / MD* Bachelor of Medicine, Bachelor of Surgery; MSc – Master of Science; *MPH* Master of Public health; *PhD* Doctor of Philosophy

For these newly established hubs, it is still too early to definitively assess their outcomes and impact on national health systems; however, the range and quality of their intermediate outputs in a relatively short space of time has been encouraging (Table 6). The open crowdsourcing calls have been a key approach that has led to the identification of over 200 projects. This approach was customised to be effective despite the bandwidth, electricity and other contextual challenges faced. The research conducted by the hubs have led to initial evidence being produced on 37 social innovations that have improved the access, quality or affordability of health services. Advocacy efforts through films and stakeholder convenings, have raised awareness of these models at international, national and district level.

**Table 5:** Elements supporting institutionalisation of social innovation within universities and nationally

Element	Description	Rationale	Practical application
1. Core hub team	A hub lead, a project manager & a project coordinator	To implement activities, build momentum and achieve sustainability.	Each hub has its own multi-disciplinary core team of a minimum of 2 full-time and 1-part time staff (Lead)
2. Strategic & Operational plan	A vision, strategic objectives and 3-year implementation plan designed to be in line with country and university priorities.	To identify key gaps and opportunities within the local health system and within the university where the hub can make a contribution, as well as to identify opportunities for partnerships and funding.	In the Malawi Hub, the hub narrowed its focus to be in line with the national priority on achieving accessible, quality primary health care in support of universal health coverage. In the Uganda Hub, a strategic advisory board has been established to guide and support the hub in achieving its strategic objectives.
3. Partnerships	Cross-university or cross-faculty partnerships Partnerships national organisations, leaders and champions.	To achieve institutionalisation of the social innovation approach, beyond the life span of the hub, within other departments, disciplines and organisations.	In the Philippines Hub, a partnership was established with the national health research agency resulting in a national award for social innovation. The Hub has further promoted social innovation at the level of the Chancellor, resulting in a university-wide identification of social innovations in health.
4. Leveraging resources	Funding, technical support or time can be leveraged from other partners/ local organisations to co-implement activities and extend impact	To supplement the initial grant funding awarded to each institution and to lower its dependence on a single funding source.	The Philippines Hub have gained additional research grant funding or as contracted consultants. The Malawi hub is leveraging academic faculty to support student research in social innovation.

### Factors that enabled or hampered the operation of university-based hubs

A cross-hub comparison of the implementation process across the five LMIC university hubs revealed the enablers and barriers experienced by the hubs. Three enablers identified for the implementation and functioning of hubs were: strong pre-existing social networks, being a part of a global network, and receiving a capacity development package. Three barriers to hub implementation and functioning were specified as: internal institutional resistance, administrative challenges and short funding cycles.

### Enablers

The first enabler that contributed to the influence the hubs have had in their countries is the presence of pre-existing relationships and strong social networks. Committed country teams with strong social capital and networks are key to bring together diverse actors across the local health system are required to build momentum at a national level. Each country team could identify champions from their own networks who could support their hub and advocate for SI. In the Philippines, the leverage of pre-existing social capital assisted in incorporating SI as a key theme in the National Health Research Council and led to the creation of a National Award for Social Innovation. In Uganda, the hub lead had a good prior working relationship with the Ministry of Health that facilitate its engagement in hub activities.

A second enabler of hub adoption and implementation was being part of a broader multi-country network. Being part of a global network has brought considerable credibility to hubs at a country-level especially as when an initiative is new, being able to show that other LMICs universities are also adopting SI in health gave legitimacy to the hub’s endeavours to engage in SI in health. Hubs have also benefited from the participation of international agencies such as TDR and PAHO in the network which have further supported credibility at country, regional and global level. Although hubs were given the opportunity to develop their own tailored individual country strategies, being part of a network assisted in fostering ongoing peer-learning, leveraging knowledge resources created by individual hubs and collaborating research publications. Hub members have come to greatly value the strong relationships and trust that have been built across individuals and the participating countries.

A third enabler of hub implementation was the provision of a capacity building package inclusive of four support modalities: training workshops, a replication package of tools and resources, technical assistance and strategic coaching. This was delivered in a phased approached based on demand. In 2017, technical assistance was in high demand, with focus on the hubs gaining theoretical and practical knowledge on SI through activity implementation (see Table 2). An intensive one-week training workshop to build university partners’ technical knowledge in SI in an experiential manner and to foster relationships between university partners. In the first year, this training was aimed at the senior academic lead responsible for the new hub and in the second year, it was aimed at project managers and co-ordinators implementing the day to day activities. A package of technical guides, templates and tools summarising the learning from 2013 to 2016 was compiled and shared with each new hub. Technical guides covered crowdsourcing contests, case study research and a protocol with data collection instruments, guides on storytelling and communication templates. This package was complimented by additional materials as identified by the ongoing needs of the hubs. Hubs received dedicated professional support in branding, communication and storytelling efforts. Throughout mentorship and strategic coaching was delivered. This mainly occurred electronically via weekly phone conversations and email support and the virtual support was also complimented by a 2 week per year period of onsite technical assistance. In 2018, as teams developed, the demand shifted to more strategic coaching and guidance, with focus on local customization and adaptation, through individual hub strategy development. Over time, as hubs became more independent, the support required and provided was reduced.

**Table 6:** Outputs of hubs in each core component area (2015–2018)

Core component	Mechanism	Output
Research	Crowdsourcing contests	6 crowdsourcing contests conducted 249 eligible projects identified across by state and non-state actors, across 48 countries.
	Case Study Research	37 case studies conducted on social innovations across 17 countries. Two WHO publications. 5 conference presentations
Community-building	Convenings and communication	6 international convenings with 200 participants from 24 countries. 4 national convenings that included policy makers. A unified web platform for sharing & communicating. 12 training workshops for social innovators in research and communication (Colombia, Malawi, Philippines, Uganda)
Storytelling	Film & media	30 social innovation case films 2 thematic social innovation films 12 promotional videos
Institutional embedding	Embedding in universities	Cross-university, cross-disciplinary student case research (Malawi) Research grants (Uganda) Student module on social innovation in health (Philippines) Award for social innovations from the University of Philippines, School of Medicine
	Embedding in national institutions	A national annual Philippine award, the Gelia Castillo Award on Research for Social Innovation in Health (GCARSIH)^a^ in partnership with the Philippines Council for Health Research and Development (Department of Science and Technology) and the Department of Health. Partnerships with National Ministries of Health on various activities – Philippines, Uganda, Malawi
	Embedding international institutions	Endorsement by the World Health Organization, Swedish International Development Cooperation Agency, UNAIDs, Pan American Health Organization.

^a^Dr. Gelia Castillo is a Sociologist and National Scientist for Research in the Philippines. She was also the vice chair for WHO COHRED – Council on Health Research and Development (1993)

### Barriers

At the same time, the hub model faced barriers to its operation in several areas. The first barrier each of many hubs had to overcome was internal institutional resistance. As the newer SI hubs were all located within an existing health faculty, such as malaria, infectious disease, clinical epidemiology, they were going against the convention of what their department ‘usually’ do. Hub members were often questioned, faced doubt or criticism for placing a focus on SI in health. Yet, this is not an uncommon reproach in response to pioneering something new. Hubs were able to overcome this resistance by positioning SI as not a new field but rather a complimentary and cross-cutting approach that can be incorporated into current work. Being able to position SI as a complimentary approach, instead of a deviation, was important to gain acceptance within the university. In Malawi and the Philippines, this positioning has assisted university leaders to start to identify additional opportunities to integrate a SI approach across the health sciences of the university. Most hubs have invested significant time to engage with faculty across the university, including senior leadership.

A second barrier faced in adopting and implementing SI hubs in universities were administrative challenges experienced when hub teams wanted to execute activities different from the norm and within shorter timeframes. The hubs in essence didn’t function like a traditional university department but more like entrepreneurial start-ups. The storytelling component of the hub required communication and film specialists to be contracted. To add a new contractor to the university system caused significant challenges especially if the contractor was based in one of the other hub countries. For field work engagements to the social innovators, staff often had to pay out of pocket to cover expenses and then wait several months for reimbursements to occur. The hiring of short-term project staff also became a significant challenge, as university hiring processes are formalised and lengthy, often causing more work for the hub staff than just putting in additional hours. As committed especially the junior staff was, working overtime as a norm and not an exception became unsustainable and a threat to staff retention.

A third barrier was the short cycles of funding support given to the hubs. Due to the funder also being dependent on its donors, most funding was provided in one-year cycles. Implementing a range of diverse activities across a short period of time, while facing administrative challenges and also contextual challenges such as electricity outages made it challenging for hubs to produce deliverables according to their set timeline.

## Discussion

There are emerging conceptual and empirical evidence that SI could help to mobilise the creation of solutions to achieving UHC in a participatory manner in diverse settings. Current literature alludes to the contribution universities who adopt SI as part of their knowledge, education and relationship-building strategy can make in addressing key challenges faced by society. It also shows that a dedicated focus within universities on SI leads to enhanced knowledge production and evidence on the concept. The adoption and implementation of SI within universities have been studied in high income country settings [15–18]. Literature on SI in healthcare refer to specific SI projects or programmes but there is limited evidence on how SI as a whole can be supported, how the local eco-system for innovation can be strengthened and the role universities can play to facilitate SI at a national level towards the progress on equitable healthcare delivery in LMICs.

This case study sets out to provide insight into how LMIC universities could play a lead role in the promoting the adoption and implementation of SI in healthcare delivery. It describes a hub model tailored for LMICs as well as the features, components, enablers and inhibitors of the hub implementation process. The five SI hubs implemented across three regions are cross-disciplinary and cross-sectoral university-based platforms, built with a shared vision to unlock SI n in local healthcare delivery systems through 1) research; 2) community building; 3) storytelling and 4) embedding SI within the institution and beyond.

SI hubs have the implication to support progress towards UHC in LMICs, to foster greater participation in health systems and to reframe the prevailing perspective of universities from elitist towards inclusive and socially conscious institutions. Within each of the five hub countries, the achievement of UCH, through the equitable delivery of quality health services at an affordable cost, is a key target of national health agendas. Evidence on how to achieve this mainly originates from externally funded research projects aiming to find answers for barriers hindering UHC. Yet, adopting an asset-based SI mindset, opens up the possibility to look for and identify already existing models that are achieving UHC at country-level, even if at a small localised scale. The work of these SI hubs has led to context-appropriate and culturally relevant SI models to be identified and studied for their potential impact or lessons they can contribute towards UCH achievement. The response from local Ministries of Health to this work conducted by the SI hubs has been very positive, especially as many of these country-based existing models, developed by non-traditional actors, were previously unknown at a central level. Ministries have shown interest in learning best-practice lessons from these models especially as their localised bottom-up nature can overcome some of the failures, especially around sustainability, these countries experience that are associated with health interventions that are developed in northern settings and implemented in a top-down manner prescribed manner. In some of the SI cases, this has moved to discussions on how SI could become adopted as part of the public health system and scaled up nationally. Beyond just a country-level, the case evidence generated by the hubs have sparked greater interest in the role SI could play in achieving UCH in both multilateral and bilateral agencies. These agencies have incorporated SI as part of their portfolios and in turn provided support for the work of the hubs and for new hubs to be developed.

The hubs moved beyond the traditional university role of theoretically and academically engaging in a subject, to playing a very practical role in the process to advance SI. True to the nature of the democratic paradigm of SI, the hubs have fostered greater social inclusion, participation and empowerment among local actors of the health system, often giving voice to previously unacknowledged actors. The hubs were able to serve as independent cross-boundary platforms, uniting actors across sectoral, disciplinary and power-hierarchy levels. This has meant that the conversation on and participation in the achievement of equitable quality health services in each nation have been more streamlined and focused as actors from academia, government, private sector and civil society have started working together in greater collaboration around specific social innovations. These platforms have become ‘one stop shops’ where policy-makers can engage with innovators and communities, researchers can develop evidence to new questions arising and private sector actors can share necessary resources to support replication or scale. The value add of university-hubs is its potential it has for affecting a culture shift in healthcare. By engaging with educators and students, and by embedding the SI approach as part of existing curricula the next generation of qualifying health professionals would be more attuned to working in a cross-boundary collaborative manner. Contrary to the predominant role universities in HIC play as proponents, and often producers, of technocratic solutions to social challenges, the key role LMIC universities are rather as eco-system enablers of SI through its advocacy efforts to various actor groups and a facilitator of this participative process.

Finally, this contextualised LMIC model of SI hubs supports reframing the university institutions as relevant, socially conscious and closer to the grassroot realities of their countries. The hubs have contributed to fostering much closer relationships between communities and the university and contributed to supporting the learning and growth of these community groups engaged in SI. As a result of this interaction, it has positioned these institutions with a strategic advantage, and it has opened up new reputational and resource opportunities for them enabling them to expand the activities. Several of country hubs have been approached by bilateral agencies due to their insight of activities happening at community level. Other national agencies have also requesting support on how to engage with social innovators and how to adopt a SI approach in their work. In a relatively short period of time, these universities have become known nationally and internationally for their work in SI. We believe this has been important in correcting some of the residual colonial dynamics that have existed between HIC and LMIC universities in the past and created a more equal the playing field [31]. The hubs have shown the competency and capacity of LMIC universities to pioneer a new field in a contextually relevant manner in country settings that are receptive and open to new players and new approaches in healthcare delivery.

This hub model and approach could hold relevance and replicability for other LMIC universities facing similar contextual conditions. Several considerations should be taken in learning from this model, at both university and country level. Starting an entrepreneurial-like endeavour such as a hub requires university leadership who are willing to take risk and have their prevailing institutional mindsets be challenged. To see the hub reach stakeholders and actors across different sectors requires the identification of support to appoint at least one person with dedicated time to developing the hub vision and strategy, while conducting ongoing advocacy. Finally, from our experience in five countries, we have seen the readiness that LMICs have to embrace SI in the area of healthcare delivery, but this involves a gradual process of transformation. With multiple competing priorities and contextual challenges, patience is required for the university hub to build relational capital and trust within local actors that could lead to sustained and durable SI at a local level.

Several limitations need to be acknowledged. This case study was conducted retrospectively, and thus depended on documentation and reflections of those involved, which may have led to focus on certain barriers and enablers while others being mentioned less often. The differences in geographical focus (regional vs national) between the hubs does hold a limitation for direct cross-country comparison. It is also noted that language barriers may have had an impact on implementation of the hubs, as the capacity building support to the hubs were provided only in English and not in Spanish or other local languages. In the LAC region, the hub had to develop a lot of their own advocacy materials as English materials were not suitable. The research was also only conducted in English.

## Conclusions

This case study shows the opportunity that reside within LMIC universities to act as eco-system enablers of SI in healthcare delivery. It highlights the possibility of establishing SI hubs, by leveraging the existing research capacity present within these institutions combined with providing the necessary start-up support over a period of time. As found in this case, university-based SI hubs can fill the evidence gap that exist on SI in healthcare delivery in an LMIC context and enhance cross-sectoral participation in support of achieving UHC.

## Data Availability

The data used for this article is available from the corresponding author on reasonable request.

## Abbreviations

BA: Bachelor of the Arts
B Pharm: Bacelor of Pharmacy
CIDEIM: Centro Internacional de Entrenamiento e Investigaciones Médicas
GSB: Graduate School of Business
HIC: High Income Countries
LMICs: Low and Middle-income Countries
LAC: Latin America and Caribbean
MBBS / MBChB / MD: Bachelor of Medicine, Bachelor of Surgery
MSc: Master of Science
MPH: Master of Public health
PAHO: Pan American Health Organization
PhD: Doctor of Philosophy
RN: Registered Nurse
SI: Social Innovation
UCT: University of Cape Town
TDR: The Special Programme for Research and Training in Tropical Diseases, cosponsored by the United Nations Children’s Fund, the United Nations development Programme, the World Bank and the World Health Organization
